# Frontline Health Workers' Perceptions of Digital Health Platforms: Qualitative Insights From the North Guwahati Block in Assam

**DOI:** 10.7759/cureus.112153

**Published:** 2026-07-06

**Authors:** Sampriti Paul, Priyanka Saha, Mintu Dewri Bharali, Achyut Chandra Baishya, Jutika Ojah

**Affiliations:** 1 Department of Community Medicine, Gauhati Medical College and Hospital, Guwahati, IND

**Keywords:** assam, ayushman bharat digital mission, braun and clarke’s framework, digital health, frontline health workers, perceptions, qualitative study, rural health, teleconsultation, user-centered design

## Abstract

Background: Digital health platforms are increasingly promoted as tools for strengthening health systems, improving access to care, and enhancing patient outcomes. In Northeast India, where difficult terrain, dispersed populations, and healthcare access challenges persist, evidence regarding frontline health workers' (FLWs') experiences with digital health platforms remains limited.

Objectives: The objective of this study is to explore the perceptions, experiences, and challenges of FLWs in relation to the adoption and use of digital health platforms in rural Assam.

Methods: A qualitative study was conducted between April and May 2026 in the North Guwahati block of Assam, covering one district hospital, one community health center, three primary health centers-health and wellness centers, and three sub-center-health and wellness centers. FLWs, including medical officers, community health officers, auxiliary nurse-midwives, Multipurpose workers, and Accredited Social Health Activists (ASHA), were selected purposively. Participants were recruited until data saturation was achieved, ensuring that no new themes emerged. Two additional interviews were reviewed after thematic redundancy to confirm saturation. Data were collected through semi-structured, in-depth interviews, which were audio-recorded, transcribed verbatim, and ​thematically analyzed following Braun and Clarke’s six-step framework. Digital health platforms included routinely used government-supported digital systems for service delivery, reporting, teleconsultation, and beneficiary tracking.

Results: Four key themes emerged: perceived benefits, operational challenges, capacity constraints, and sustainability concerns. While FLWs appreciated the improved access, convenience, and documentation offered by digital platforms, participants reported persistent issues related to poor internet connectivity, duplication of reporting systems, inadequate training, increased workload, and limited interoperability between digital platforms.

Conclusion: FLWs perceive digital health platforms as beneficial for patient care but face significant operational, capacity, and sustainability challenges. Strengthening infrastructure, ensuring interoperability, providing continuous training, and addressing equity concerns are essential for the effective and sustainable scale-up of digital health initiatives in rural India.

## Introduction

The integration of digital technologies into healthcare systems has been increasingly recognized as a transformative approach to addressing persistent gaps in access, efficiency, and quality of healthcare delivery worldwide. The World Health Organization (WHO) defines digital health as the use of digital, mobile, and wireless technologies to support the achievement of health objectives [1]. The Global Strategy on Digital Health 2020-2025 emphasizes digital health as an essential enabler of Universal Health Coverage (UHC) and Sustainable Development Goals (SDGs), highlighting its role in strengthening health systems, improving access to care, and ensuring equitable health outcomes [2].

In low- and middle-income countries (LMICs), including India, digital health platforms have emerged as potential solutions to bridge disparities in healthcare access, particularly in rural and underserved areas [3,4]. Teleconsultation, electronic health records, decision-support systems, and mobile health applications are increasingly being used to overcome geographical barriers, reduce patient out-of-pocket expenditure, and improve continuity of care. These tools are particularly relevant in resource-constrained health systems characterized by inadequate specialist availability, fragmented referral systems, and weak health information infrastructures [5].

India has made significant strides in advancing digital health initiatives. The launch of the National Digital Health Mission (NDHM) in 2020, now known as the Ayushman Bharat Digital Mission (ABDM), marked a landmark effort to digitize healthcare through unique health IDs, electronic health records, and interoperable systems [6]. Complementing this, e-Sanjeevani, the government’s flagship telemedicine platform, has provided more than 200 million teleconsultations since its inception, demonstrating the potential of digital health to extend its reach even during the COVID-19 pandemic [7].

Despite these advancements, challenges remain in their implementation, especially at the primary and secondary care levels. These include poor Internet connectivity, limited digital literacy, multiple parallel reporting systems, and inadequate integration with existing government portals, such as the Reproductive and Child Health (RCH) and Non-Communicable Disease (NCD) portals [8,9]. These challenges may be particularly pronounced in Northeast India, where geographical barriers, variable internet connectivity, dispersed populations, and resource constraints can affect the implementation and sustainability of digital health initiatives.

While patients are the ultimate beneficiaries of digital health, frontline health workers (FLWs), including medical officers (MOs), community health officers (CHOs), auxiliary nurse midwives (ANMs), and multipurpose workers (MPWs), are the primary implementers. Their acceptance, use, and adaptation to digital health platforms determine the feasibility and sustainability of these innovations [10]. In this study, “digital health platforms” refer broadly to the tools commonly used in India’s public health system, including teleconsultation services (e.g., e-Sanjeevani), digital reporting portals (such as the RCH and NCD portals), the Health Management Information System (HMIS), NIKSHAY, U-WIN and newer applications linked to the Ayushman Bharat Digital Mission (ABDM), such as the ABHA health ID and digital health records. Together, these platforms represent the ecosystem of digital health interventions encountered by FLWs in rural Assam.

FLWs are the backbone of India’s health system, especially under the Ayushman Bharat initiative, which envisions health and wellness centers (HWCs) as the hub of comprehensive primary care [11]. The introduction of new technologies directly influences workflows, reporting responsibilities, and patient interactions. Existing literature suggests that FLWs’ perceptions shape technology adoption: a positive attitude can accelerate uptake, while negative experiences, such as workload burden, inadequate training, or lack of technical support, can lead to resistance or abandonment [12,13].

Several studies from other parts of India have highlighted the benefits of digital tools, including improved documentation, patient follow-up, and better access to specialists [14,15]. However, these studies also reported barriers such as duplication of data entry, connectivity failures, and misalignment with existing reporting structures [16,17]. Importantly, very few studies have focused on the northeastern region of India, despite its distinct health system challenges, including difficult terrain, lower health infrastructure density, and significant rural-urban disparities.

Increasingly, the success of digital health interventions is being linked to the principles of user-centered design (UCD), which emphasize the active involvement of end-users throughout the design, implementation, and evaluation of digital systems. UCD recognizes that technologies are more likely to be adopted and sustained when they align with the needs, workflows, capabilities, and contextual realities of intended users [18]. In the context of public health systems, FLWs represent the primary users of many digital platforms and play a critical role in translating technological innovations into service delivery. Understanding their experiences and perspectives is therefore essential for identifying barriers to adoption, improving usability, and ensuring that digital health solutions are responsive to local healthcare needs.

There is limited evidence on how FLWs in Assam perceive digital health platforms, despite their central role in implementing these technologies and connecting communities with the health system. Understanding their experiences can provide valuable insights into barriers, facilitators, and system-level challenges affecting digital health adoption. Therefore, this study explored the perceptions of FLWs regarding digital health platforms in North Guwahati block, Assam, using a qualitative approach to capture their experiences and inform the future integration and scale-up of digital health initiatives in similar resource-constrained settings.

## Materials and methods

Methodology

This study adopted a qualitative exploratory research design to gain in-depth insights into the perceptions and experiences of FLWs regarding digital health platforms and was considered appropriate given the study’s objective. The study was guided by a constructivist paradigm, acknowledging that realities are socially constructed and best understood through participant narratives [19].

The study was conducted in North Guwahati block, Kamrup district, Assam. The North Guwahati block comprises a total of 33 public health facilities across different levels of care. For the present study, one district hospital (DH), one community health center (CHC), three primary health centers (PHCs), and three Ayushman Arogya Mandir-Sub Centers (AAM-SCs) were purposively selected to ensure representation across all tiers of the healthcare delivery system and to capture diverse experiences of FLWs involved in digital health platform implementation. The study population comprised FLWs involved in the delivery of primary healthcare services and implementation of digital health platforms in public health facilities of North Guwahati block, Assam. FLWs were defined as community- and facility-level healthcare providers who serve as the first point of contact with the health system and are directly engaged in preventive, promotive, and basic curative services. Accordingly, the study included accredited social health activists (ASHAs), ANMs, CHOs, MPWs, and MOs.

A purposive sampling strategy was employed to ensure representation across facility levels and cadres. Inclusion criteria were: (i) currently employed in the facility for at least six months, (ii) directly engaged with digital health platforms, and (iii) willingness to participate. FLWs with no prior experience or exposure to digital health platforms were excluded from the study. Staff on extended leave or administrative postings with no direct patient interaction were also excluded. Thirty participants were interviewed: three CHOs, eight ANMs, five MOs, eight ASHAs, and six MPWs. This sample size was considered sufficient to reach data saturation [20]. Two additional interviews were reviewed after thematic redundancy was observed to confirm data saturation. Data were collected between April 2026 and May 2026 using in-depth interviews. A semi-structured interview guide was developed after reviewing existing literature [3-5] and in consultation with subject experts. The semi-structured interview guide explored participants’ roles and responsibilities in routine service delivery, experiences with digital health platforms (including perceived benefits, ease of use, and challenges), perceptions of patient and community responses, barriers and facilitators to digital health adoption, and recommendations for improving system design and sustainability.

Interviews were conducted in Assamese and English, depending on participant preference. Each interview lasted 30-45 minutes and was conducted in a private setting within the facility to ensure confidentiality and comfort. All interviews were audio-recorded with participants' consent and supplemented by field notes documenting non-verbal cues and contextual observations.

All interviews were transcribed verbatim by the research team. To ensure transcription accuracy, each transcript was independently reviewed against the original audio recording by a second researcher. Any discrepancies, unclear sections, or omissions were discussed and resolved through consensus. The verified transcripts were subsequently translated into English. To preserve meaning and contextual nuances, translated transcripts were reviewed by a second bilingual researcher. All transcripts were anonymized, with codes assigned to participants (e.g., CHO-PHC1, MO-CHC). Data were stored securely in password-protected folders accessible only to the research team.

Data were analyzed using Braun and Clarke's reflexive thematic analysis approach [21]. Familiarization with the data was achieved through repeated reading of transcripts. Initial coding was undertaken by two members of the research team to facilitate reflexive engagement with the data and encourage diverse interpretive perspectives. Rather than seeking inter-coder agreement or measuring coding reliability, coding discussions were used to critically reflect on emerging patterns, refine interpretations, and support theme development. Themes were developed iteratively through ongoing engagement with the data and regular analytic discussions among the research team. Reflexive notes were maintained throughout the analytic process. The process involved six iterative steps: (i) Familiarization: Reading and re-reading transcripts to gain an overall understanding; (ii) Initial coding: Generating codes line-by-line using Taguette software. Two researchers coded transcripts independently; (iii) Searching for themes: Codes were grouped into broader categories reflecting patterns in perceptions; (iv) Reviewing themes: Categories were refined and collapsed into overarching themes; (v) Defining and naming themes: Clear definitions were developed for each theme to ensure conceptual clarity; (vi) Reporting: Verbatim quotes were selected to illustrate key themes, ensuring representation across cadres and facility levels.

Disagreements in coding were resolved through discussion, and reflexivity was maintained by documenting researcher assumptions and perspectives throughout the process. Ethical clearance was obtained from the Institutional Ethics Committee, Gauhati Medical College and Hospital. Administrative approval was also taken from the District Health Society. Written informed consent was obtained from all participants prior to data collection. Participants were informed about voluntary participation, the right to withdraw at any point, and confidentiality of responses. Identifiers were removed during analysis and reporting.

## Results

A total of 30 FLWs were interviewed across selected public health facilities in North Guwahati block. The participants represented multiple cadres and facility levels (Table 1).

**Table 1:** Sociodemographic and professional characteristics of study participants (N = 30). Data are presented as frequency (n) and percentage (%). Prior training refers to formal orientation sessions, government-supported cascade trainings, workshops, or platform-specific training related to digital health systems used in routine service delivery. ASHA: Accredited social health activist; ANM: auxiliary nurse midwife; MPW: multipurpose worker; CHO: community health officer; MO: medical officer; CHC: community health center; DH: district hospital

Characteristics	Category	Frequency (n)	Percentage (%)
Age (years)	21–30	6	20
	31–40	11	36.7
	41–50	13	43.3
Sex	Female	18	60
	Male	12	40
Cadre of FLW	ASHA	8	26.7
	ANM	8	26.7
	MPW (Male/Female)	6	20
	CHO	3	10
	MO	5	16.7
Educational Qualification	Up to Class 10	8	26.7
	Class 12	10	33.3
	Graduate & above	12	40
Type of Posting	Health & Wellness Center (Sub-center)	10	33.3
	Health & Wellness Center (PHC)	12	40
	CHC/DH	8	26.7
Prior Training in Digital Health Platforms	Yes	22	73.3
	No	8	26.7
Average Daily Use of Digital Platforms for Work	< 1 hour	8	26.7
	1–2 hours	22	73.3

A total of 30 FLWs participated in the study. The largest proportion of participants were aged 41-50 years (n=13, 43.3%), followed by those aged 31-40 years (n=11, 36.7%) and 21-30 years (n=6, 20.0%). Female participants constituted 60.0% (n=18), while male participants accounted for 40.0% (n=12).

In terms of cadre distribution, ASHAs (n=8, 26.7%) and ANMs (n=8, 26.7%) represented the largest groups, followed by MPWs (n=6, 20.0%), MOs (n=5, 16.7%), and CHOs (n=3, 10.0%).

Regarding educational qualification, 40.0% (n=12) of participants were graduates or above, 33.3% (n=10) had completed higher secondary education (Class 12), and 26.7% (n=8) had studied up to Class 10.

Most participants were posted at HWCs, with 40.0% (n=12) working at HWC-PHCs and 33.3% (n=10) at HWC-Sub Centers, while 26.7% (n=8) were posted at CHC/DH-level facilities.

Nearly three-fourths of participants (n=22, 73.3%) reported having received prior training in digital health platforms, whereas 26.7% (n=8) had not received any formal training. Similarly, 73.3% (n=22) reported using digital platforms for 1-2 hours daily as part of their routine work, while 26.7% (n=8) used them for less than one hour per day.

**Figure 1:**
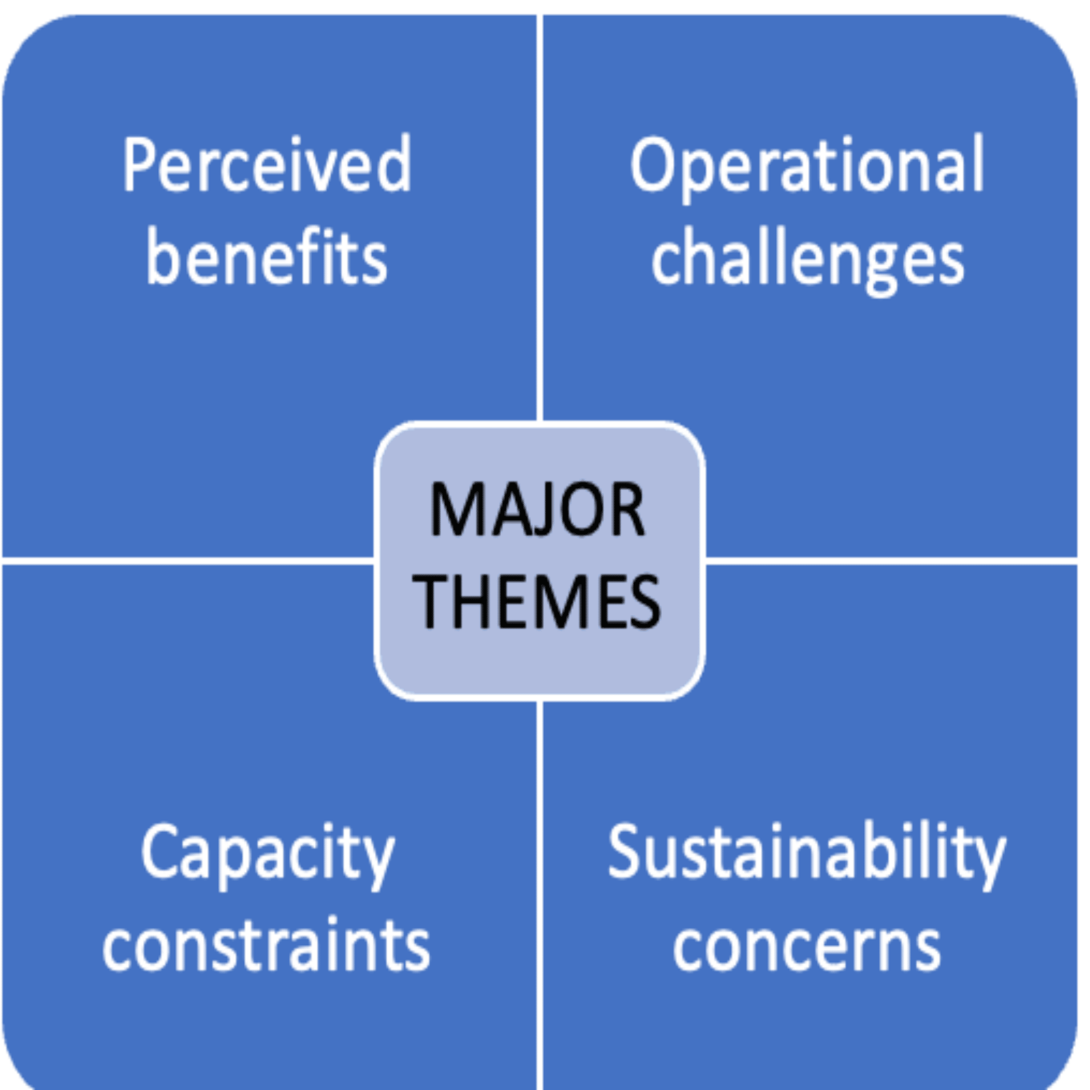
Thematic framework of frontline health workers’ perceptions of digital health platforms. Created using Microsoft Word (SmartArt and Shapes tools)

Figure 1 illustrates the four major themes that emerged from the thematic analysis of in-depth interviews with FLWs: perceived benefits, operational challenges, capacity constraints and sustainability concerns. These themes represent the key domains shaping frontline experiences with digital health platforms in the study setting.

**Figure 2:**
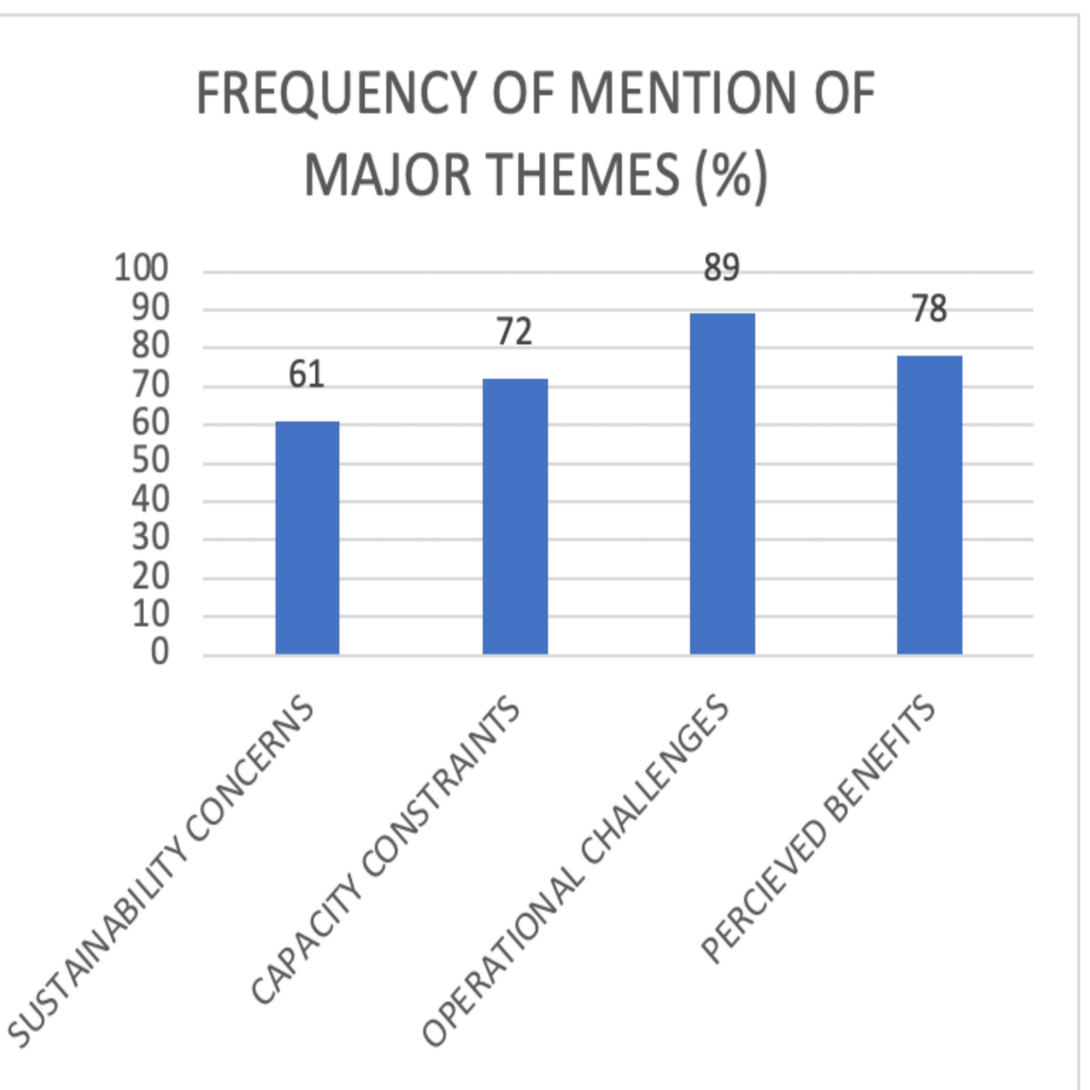
Frequency of mention of major themes reported by frontline health workers. Values represent the percentage (%) of participants (N=30) who mentioned each theme during the interviews. Values represent the proportion of participants who mentioned each theme during interviews. These frequencies are presented descriptively to illustrate relative prominence and should not be interpreted as statistical measures of importance.

Figure 2 presents the proportion of participants who reported each major theme during in-depth interviews. Operational challenges were the most frequently mentioned theme (89%, n=27), followed by perceived benefits (78%, n=23), capacity constraints (72%, n=22), and sustainability concerns (61%, n=18).

Table 2 presents the thematic synthesis of FLWs' perceptions regarding digital health platforms, organized into four major themes and corresponding subthemes. Overall, while participants acknowledged substantial benefits, particularly in improving access to specialist care, reducing patient costs, and enhancing documentation, these advantages were consistently counterbalanced by significant operational and systemic challenges. Operational issues, especially poor network connectivity and duplication of reporting across multiple platforms, emerged as the most frequently cited concerns, highlighting gaps in infrastructural readiness and system integration. Capacity-related constraints, including inadequate training, increased workload, and role-related tensions, further influenced the usability and acceptance of digital tools among health workers. In addition, participants expressed concerns regarding the long-term sustainability of digital health initiatives, citing high operational costs, lack of interoperability between platforms, and the perception of such interventions as externally driven rather than embedded within routine health systems.

**Table 2:** Themes, subthemes, and illustrative quotes on perceptions of FLWs regarding digital health platforms. ANM: auxiliary nurse midwife; MPW: multipurpose worker; CHO: community health officer; MO: medical officer; CHC: community health center; DH: district hospital; PHC: primary health center; FLWs: frontline health workers

Theme	Subtheme	Key Findings	Verbatims (Cadre, Facility)
Perceived Benefits of Digital Health Platforms	Improved access to specialists	Teleconsultation reduced dependency on physical referrals and enabled specialist advice at peripheral facilities, enhancing confidence in primary care.	“Earlier we had to refer patients outside; now they can consult specialists directly.” (MO, CHC)
	Reduced patient cost & inconvenience	Participants perceived that digital platforms reduced travel costs, wage loss, and inconvenience for patients, particularly in riverine and peri-urban areas.	“She saved almost 500 rupees… she was very happy.” (CHO, PHC)
	Better documentation and continuity of care	Digital systems were perceived to improve record-keeping and facilitate follow-up, particularly in immunization services.	“Now the system itself reminds us about follow-up and dates.” (ANM, PHC)
Operational Challenges in Everyday Use	Poor network connectivity	Unreliable internet was the most frequently reported barrier, often disrupting patient consultations and service flow.	“Many times everything is ready, but the internet doesn’t work.” (CHO, PHC)
	Gaps between intended design and actual use of digital systems	Although ABHA IDs are routinely generated, comprehensive electronic health records are not maintained, limiting continuity of care and reducing the functional value of digital health systems.	“ABHA ID is made, but we don’t maintain full patient records digitally.” (CHO, PHC)
	Selective, program-specific utilization of platforms.	Digital platforms such as U-WIN are predominantly used for immunization, with limited or no use for other services like ANC and NCD care.	“We mainly use it for immunization work; other services are not followed like that.” (ANM, PHC)
	Absence of integrated reminder systems for ANC and NCD services	Unlike immunization, no automated reminder or due-list system exists for ANC visits or NCD follow-up, leading to missed opportunities for longitudinal care.	“There is no reminder for ANC or BP patients like immunization due list.” (MO, PHC)
	Underutilization of digital outputs	Even when due lists are generated (e.g., through U-WIN), they are not consistently integrated into routine workflows, resulting in underutilization of available digital functionalities.	“Due list comes, but is not always used in daily work.”(ANM, SC)
	Duplication of reporting	Coexistence of multiple portals and paper registers increased clerical burden and reduced perceived efficiency of digitalization.	“We enter data in portals and still maintain paper registers.” (ANM, PHC)
	Dependence on patient phone numbers	Lack of personal mobile phones, frequent number changes, and shared devices led to missed follow-ups and exclusion of vulnerable groups.	“Many villagers don’t have mobiles; follow-up gets missed.” (MO, DH)
Capacity and Role-Related Constraints	Inadequate training	One-time, brief trainings were insufficient to build confidence in using advanced features of digital platforms.	“Training happened once; later when problems come, no one helps.” (ANM, CHC)
	Increased workload	Digital documentation added to administrative work, reducing time for direct patient care.	“I spend more time typing than attending to patients.” (ANM, DH)
	Role-identity conflict	Medical officers perceived digital data entry as clerical work, affecting professional satisfaction and role clarity.	“Doctors end up doing work meant for data staff.” (MO, DH)
	Limited ownership of digital platforms (ASHAs)	While ASHAs are actively involved in mobilization, beneficiary identification, and facilitation of digital services, their engagement remains indirect, with limited control over data entry and platform use due to structural and capacity constraints.	“We collect the information, but entry is done by ANM/CHO.” (ASHA, SC)
Sustainability and System-Level Concerns	Multiple unlinked platforms in routine work	FLWs have to work across several separate portals that are not connected, making routine service delivery fragmented and time-consuming.	“Every year a new portal comes, but old ones never go.” (CHO, PHC)
	Lack of continuity in use of digital systems	Digital platforms are introduced but not consistently continued or fully adopted in routine workflows, leading to partial or irregular use.	“Sometimes we use it, sometimes we go back to registers.” (ANM, PHC)
	No single platform for comprehensive patient care	Different services (immunization, ANC, NCD) are managed on separate systems, preventing a unified view of patient information.	“For each work there is a different system, nothing is together.” (CHO, SC)
	Perceived difficulty in long-term continuation	FLWs feel that digital systems require continuous resources (internet, devices, human resources), which may be difficult to sustain in routine settings.	“Such systems are difficult to continue regularly.” (MO, DH)

**Figure 3:**
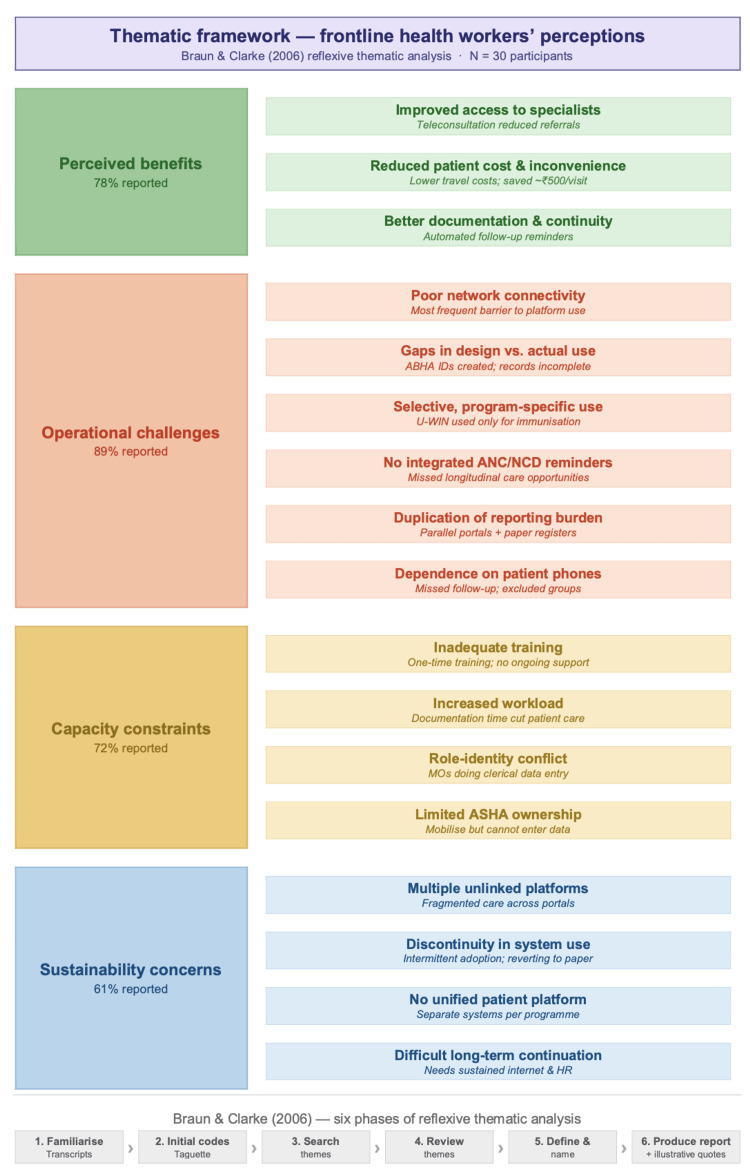
Detailed thematic framework of frontline health workers' perceptions of digital health platforms in North Guwahati Block, Assam. Created using SmartArt in Microsoft Word through Braun and Clarke's reflexive thematic analysis approach [21].

Figure 3 presents the detailed thematic framework developed through Braun and Clarke's reflexive thematic analysis approach. The figure illustrates the relationships between the four overarching themes and their corresponding subthemes.

Table 3 presents the cross-cutting analytical insights derived from the thematic analysis, highlighting broader systemic and contextual dimensions influencing the implementation of digital health platforms. The findings underscore important equity concerns, as reliance on mobile phone-based systems risks excluding vulnerable populations such as women, the elderly, and individuals from lower socioeconomic backgrounds who may lack consistent access to personal devices.

**Table 3:** Cross-cutting analytical insights from thematic analysis.

Analytical Lens	Finding
Equity	Phone-dependent systems risk excluding women, elderly, and poorer households.
System Readiness	Motivation exists, but infrastructure and integration lag behind
Workforce Dynamics	ASHAs function primarily as facilitators with limited ownership of digital tools, reflecting structural hierarchy in digital health implementation.
Implementation Reality	Digital health is experienced as both empowering and burdensome.

## Discussion

This qualitative study explored the perceptions of FLWs regarding digital health platforms in a rural block of Assam, revealing a complex interplay of perceived benefits and systemic challenges. The findings highlight that while digital health platforms are recognized as valuable tools for improving access and documentation, their implementation is constrained by infrastructural, operational, and workforce-related barriers.

Participants in this study reported that digital health platforms improved access to specialist care, reduced patient costs, and enhanced continuity of care through better documentation and follow-up. These findings are consistent with emerging evidence from low- and middle-income countries, where digital health interventions, particularly telemedicine, have been shown to improve healthcare access and reduce geographic barriers to care [22,23].

A systematic review by Kruse et al. demonstrated that telehealth interventions significantly enhance patient satisfaction and reduce travel-related costs, especially in rural settings [24]. Similarly, digital health tools have been associated with improved adherence to treatment protocols and better tracking of chronic conditions through automated reminders and electronic records [25]. The present study reinforces these benefits in the context of rural Assam, suggesting that digital platforms can strengthen primary care delivery when effectively implemented.

Despite these benefits, operational challenges, particularly poor internet connectivity and duplication of reporting, emerged as the most prominent concerns. These findings reflect persistent infrastructural gaps in digital health implementation, especially in rural and geographically challenging regions. Studies from sub-Saharan Africa and South Asia have similarly identified unreliable internet connectivity and inadequate digital infrastructure as key barriers to effective digital health deployment [26,27].

The issue of parallel reporting systems, where digital platforms coexist with paper-based registers, has been widely documented as a source of inefficiency and “digital fatigue” among health workers [28]. Lack of interoperability between platforms further exacerbates this problem, leading to redundancy and reduced perceived usefulness of digital tools. These findings underscore the need for integrated health information systems that streamline workflows rather than adding to existing burdens.

Capacity-related challenges, including inadequate training and increased workload, were also prominent in this study. Participants reported that one-time training sessions were insufficient to build confidence in using digital platforms, highlighting the need for continuous capacity-building approaches. Similar findings have been reported in other LMIC settings, where ongoing mentorship and supportive supervision have been identified as critical enablers of digital health adoption [29].

Increased administrative workload emerged as a significant concern, with participants noting that digital documentation often reduced time available for patient care. This aligns with previous research indicating that poorly integrated digital systems can increase cognitive and clerical burden, thereby affecting healthcare provider efficiency and job satisfaction [30]. An important finding of this study was the perception among MOs that digital data entry constituted clerical rather than clinical work. This highlights that digital health implementation is not merely a technological intervention but also a workforce and workflow issue. Poor alignment between professional roles and digital responsibilities may affect motivation, job satisfaction, and long-term adoption of digital platforms.

One of the most significant findings of this study was the limited ownership of digital platforms among ASHAs. Although ASHAs played an essential role in beneficiary identification, mobilization, and facilitation of services, they had limited direct interaction with digital platforms and little control over data entry processes. This finding highlights structural hierarchies within digital health implementation and raises important questions regarding digital inclusion, empowerment, and equitable participation of FLWs in digital health systems.

This observation is consistent with global literature on community health workers, which suggests that while digital tools can enhance their effectiveness, lack of access, training, and decision-making authority often limits their meaningful engagement [31,32]. It is possible that gender-related factors may influence digital platform engagement among ASHAs, who are predominantly women; however, this study did not specifically explore gendered barriers and therefore this should be interpreted as a potential implication rather than a direct finding.

The findings also highlight important equity concerns, particularly the reliance on mobile phone-based systems for patient registration and follow-up. Participants noted that individuals without access to personal mobile phones-often women, elderly persons, and those from lower socioeconomic backgrounds-were at risk of exclusion. This reflects the broader “digital divide” observed in many LMICs, where disparities in access to technology can exacerbate existing health inequities [33].

Evidence suggests that digital health interventions may unintentionally widen gaps in access if not designed with an equity lens [34]. Therefore, alternative mechanisms, such as shared devices, offline functionalities, and community-based support, must be considered to ensure that digital health platforms do not exclude vulnerable populations.

Concerns regarding financial sustainability and fragmentation of digital platforms were also prominent in this study. Participants perceived digital health initiatives as externally driven and project-based, raising doubts about their long-term integration into routine health systems. Similar challenges have been reported globally, where many digital health interventions fail to scale beyond pilot phases due to lack of sustained funding and institutional ownership [35].

The lack of interoperability between platforms further undermines sustainability, as multiple standalone systems increase complexity and reduce efficiency. The concept of “digital health ecosystems,” which emphasizes integration and interoperability, has been proposed as a solution to these challenges [32]. The findings of this study support the need for a unified, government-owned digital architecture that aligns with existing workflows and reporting systems.

The findings of this study have several important implications. First, strengthening digital infrastructure, particularly reliable internet connectivity, is essential for effective implementation. Second, capacity-building efforts must move beyond one-time training to include continuous support and mentorship. Third, digital platforms should be designed to integrate seamlessly with existing workflows, minimizing duplication and administrative burden.

Importantly, there is a need to enhance the role of ASHAs in digital health systems by improving their access to devices, training, and platform ownership. Finally, policymakers must adopt an equity-focused approach to ensure that digital health interventions are inclusive and accessible to all population groups.

A major strength of this study is its inclusion of diverse cadres of FLWs across multiple facility levels, providing a comprehensive view of frontline perspectives. The use of thematic analysis with triangulated coding enhanced credibility. However, the study is limited to one block in Assam, which may affect transferability to other regions. Additionally, perspectives of patients and higher-level administrators were not captured, which could provide complementary insights. Further research is needed to assess patient experiences alongside FLW perspectives and to evaluate the cost-effectiveness of digital platforms. Comparative studies between integrated and stand-alone digital systems would provide valuable policy guidance.

Policy implications and recommendations

Structured Digital Capacity Building for Existing FLWs

Training for digital health platforms in India has largely been one-time, cascade-based, and insufficiently hands-on. Workers have reported confidence collapsing the moment a technical problem arose post-training. Recommendations should advocate for a competency-tiered training model: foundational digital literacy for ASHAs and MPWs, intermediate platform navigation for ANMs and CHOs, and advanced troubleshooting and data interpretation for MOs. Training must be iterative, not event-based - quarterly refreshers embedded into existing review meetings such as block-level monthly review meetings would impose no additional burden on the calendar. Peer-learning circles, where digitally confident workers mentor others within the same facility, have shown effectiveness in similar LMIC contexts and cost virtually nothing to institutionalize.

Dedicated Digital Health Handholding Cadre at the Block Level

A practical solution for India, given existing HR constraints, is the creation of a Block Digital Health Facilitator role, one per block, drawn either from existing data entry operators with an enhanced scope of work, or as a new contractual position under the National Health Mission (NHM) framework. This person would provide real-time troubleshooting support, conduct on-site platform orientation for newly posted staff, coordinate with state NIC or IT cells, and track platform utilization metrics. The cost-to-impact ratio of such a role would be highly favorable relative to the systemic losses caused by platform non-use or data duplication.

Development of a Unified, Interoperable Digital Health Dashboard

U-WIN for immunization, RCH portal for maternal health, NCD portal for non-communicable diseases, NIKSHAY for tuberculosis, HMIS for aggregate reporting, all functioning in silos, all demand separate data entry, and none offers a composite view of a patient or population. India's ABDM architecture already provides the interoperability backbone through the ABHA health ID, but the last-mile integration at the facility level has not happened yet.

A Unified Frontline Health Dashboard, accessible at sub-center, PHC, and CHC levels, should consolidate the following in a single interface: immunization due lists and coverage rates, ANC registration and follow-up status, NCD screening and hypertension/diabetes follow-up, TB notification and treatment outcomes, and HMIS monthly reporting indicators. This dashboard should surface automated due lists, flag missed visits, and generate facility-level performance snapshots without requiring workers to navigate multiple portals.

AI Integration into the Unified Dashboard for Predictive and Decision Support

Future research may explore the potential role of advanced analytics, decision-support tools, and artificial intelligence within integrated digital health systems. However, such innovations require rigorous evaluation, governance mechanisms, ethical safeguards, and resource investments before implementation at scale.

Voice-based data entry in regional languages: given the digital literacy constraints in our study, AI-powered voice interfaces in Assamese would dramatically lower the barrier to data entry for ASHAs and MPWs, removing the dependency on typing skills entirely. The technology exists; deployment in public health platforms simply requires political and programmatic will.

NCD risk stratification at point of care: CHOs conducting wellness visits at HWCs could use an AI-assisted clinical decision support tool that, based on entered vitals and history, flags patients requiring further investigation or specialist referral. This directly addresses the over-reliance on specialist teleconsultation for cases that could be triaged locally.

Infrastructure-First Approach: Connectivity as a Prerequisite, Not an Assumption

No digital health recommendation can be meaningful without confronting the infrastructure gap as the present study names as the single most frequently cited barrier - 89% of participants reported operational challenges, with poor connectivity at the forefront. The rollout of digital mandates by national and state programs must be preceded by connectivity audits at the facility level. Where fiber or 4G connectivity is unavailable, offline-first application design, where data is entered locally and synced automatically when connectivity is restored, must be the standard, not the exception. Several states, including Odisha and Rajasthan, have piloted offline-capable versions of health applications; Assam's NHM should formally adopt this as a technical requirement for any new platform procurement.

Equity-Proofing Digital Health: Addressing Exclusion of the Most Vulnerable

The study highlights the risk of digital exclusion among beneficiaries who lack personal mobile phones, share devices, or frequently change contact numbers. As digital platforms increasingly rely on phone-based communication and authentication, such individuals may be disproportionately disadvantaged. Future digital health programs should incorporate inclusive design features, including alternative contact mechanisms, community-supported follow-up pathways, and flexible beneficiary identification systems. Any use of persistent digital identifiers should be accompanied by robust safeguards for privacy, informed consent, and data security. Additionally, digital decision-support systems should retain provisions for manual review and override by FLWs to ensure that vulnerable or high-risk individuals are not overlooked because of incomplete or inaccurate digital records. Such measures can help balance technological efficiency with equity, accountability, and patient-centered care.

Institutionalizing Reflexive Feedback Loops between Workers and System Designers

Finally, and cutting across all of the above, our findings underscore that FLWs possess highly practical, ground-level insight into what works and what does not - insight that rarely reaches platform designers or state program managers. A formalized feedback mechanism, a simple quarterly digital feedback form embedded within the dashboard itself, reviewed by block and district program officers, would create a continuous improvement loop that keeps platform evolution anchored in frontline reality rather than policy assumption.

These recommendations move from the immediate and implementable - training, a block-level facilitator, platform rationalization, to the strategic and transformative, a unified AI-integrated dashboard, voice interfaces, predictive analytics.

Limitations

This study has several limitations. First, it was conducted in a single block of Assam, which may limit the transferability of the findings to other geographical and health system contexts. Second, the study explored the perspectives of FLWs and did not include patients, caregivers, program administrators, or policymakers, whose views may provide additional insights into digital health implementation. Third, as the data were based on self-reported experiences, there is a possibility of social desirability bias, whereby participants may have modified their responses due to professional or organizational considerations. Finally, although rigorous procedures were followed for transcription, translation, and verification of interview data, subtle linguistic and contextual nuances may have been lost during the translation process. These limitations should be considered when interpreting the findings, and future research may benefit from including a broader range of stakeholders and study settings.

## Conclusions

This study found that FLWs perceived digital health platforms as beneficial for improving access to care and documentation, but significant barriers related to infrastructure, training, workload, interoperability, and sustainability remain. Strengthening connectivity, continuous capacity building, and integrated digital systems will be critical for ensuring equitable and sustainable digital health implementation in rural India.
